# A systematic computational analysis of the endosomal recycling pathway in glioblastoma

**DOI:** 10.1016/j.bbrep.2024.101700

**Published:** 2024-04-11

**Authors:** Luke J. Joyce, Andrew J. Lindsay

**Affiliations:** Membrane Trafficking and Disease Laboratory, School of Biochemistry & Cell Biology, Biosciences Institute, University College Cork, Cork, T12 YT20, Ireland

**Keywords:** Endosomal recycling pathway, Glioblastoma, Low-grade glioma, Receptor tyrosine kinases, EGFR, LASSO cox regression analysis

## Abstract

Glioblastoma (GBM) is the most common and aggressive brain cancer in adults. The standard treatment is brutal and has changed little in 20 years, and more than 85% of patients will die within two years of their diagnosis. There is thus an urgent need to identify new drug targets and develop novel therapeutic strategies to increase survival and improve quality of life. Using publicly available genomics, transcriptomics and proteomics datasets, we compared the expression of endosomal recycling pathway regulators in non-tumour brain tissue with their expression in GBM. We found that key regulators of this pathway are dysregulated in GBM and their expression levels can be linked to survival outcomes. Further analysis of the differentially expressed endosomal recycling regulators allowed us to generate an 8-gene prognostic signature that can distinguish low-risk from high-risk GBM and potentially identify tumours that may benefit from treatment with endosomal recycling inhibitors. This study presents the first systematic analysis of the endosomal recycling pathway in glioblastoma and suggests it could be a promising target for the development of novel therapies and therapeutic strategies to improve outcomes for patients.

## Highlights

• Regulators of the endosomal recycling membrane trafficking pathway are dysregulated in glioblastoma.
• An 8-gene endosomal recycling prognostic signature can distinguish low-risk from high-risk glioblastoma.
• The endosomal recycling pathway represents a novel target for developing anticancer therapies.

## 1 Introduction

Glioblastoma (GBM) is a devastating adult brain cancer with high rates of recurrence and resistance to treatment. More than a quarter of a million people worldwide develop brain cancer each year, and the incidence of GBM is increasing due to aging populations [1]. It is a low survival cancer; without treatment average survival is 3 months and this increases to 10–13 months with treatment. The standard of care, maximal surgical resection followed by radiation and chemotherapy with temozolomide, is brutal and has not evolved since it was established almost 20 years ago [2]. There is a clear unmet need to develop new and more effective therapies to treat people with GBM, in order to increase survival rates and quality of life.

One of the characteristics of GBM that makes it so difficult to treat is the very high degree of inter- and intra-tumoral heterogeneity, with the broad range of genetic mutations between patients affecting prognosis and response to therapy [3]. GBM was the first cancer type that was comprehensively analysed by genomics methods, and careful analysis revealed that the epidermal growth factor receptor (EGFR) gene is amplified or mutated in 40–60% of GBM cases [4,5]. EGFR is a member of the ERBB family of receptor tyrosine kinases (RTKs), which also includes HER2, HER3 and HER4. It is a cell surface receptor that regulates signalling pathways which control cell division and survival, and its aberrant activation has been associated with a number of cancers, including lung adenocarcinoma, breast and head and neck cancers. There are several monoclonal antibody and small molecule therapies that specifically target EGFR and are in use in the clinic. A few EGFR-targeting tyrosine kinase inhibitors (TKIs) have been tested in clinical trials to treat GBM, but the results have been disappointing with no improvement in clinical outcome observed [6]. A third generation EGFR TKI called osimertinib holds greater promise as it can efficiently cross the blood-brain barrier and is currently undergoing clinical trials [7,8].

RTKs are continuously internalised from the plasma membrane into organelles called early endosomes by a process called endocytosis, which affects the specificity and duration of their signalling. From the early endosome they are either sent to lysosomes for degradation or are returned to the plasma membrane to be re-used. This return pathway is called the endosomal recycling pathway and is the main cellular mechanism for controlling the composition of the plasma membrane. A typical cell turns over the entire contents of its plasma membrane every 2 hours [9]. We and others have reported that genes encoding regulators of endosomal recycling are frequently mutated, amplified, overexpressed or deleted in many cancers, including glioblastoma [10,11]. This can lead to hyperactivation of endosomal recycling and cause an imbalance in the level of RTKs and other clinically relevant proteins at the cell surface, resulting in a consequent upregulation of cell proliferation and motility signals.

Furthermore, we have recently shown that inhibition of endosomal recycling in breast cancer cells with small molecule inhibitors leads to a reduction in total cellular HER2 and HER3 protein levels. We reported that blocking the recycling of these receptors results in their diversion to lysosomes where they are degraded. We also showed that endosomal recycling inhibitors synergise with HER2-targeting therapies, in both drug sensitive and drug resistant HER2-positive breast cancer [12]. Based on this and other findings from our laboratory [13], we proposed that the endosomal recycling pathway represents a novel and underexploited target for developing anticancer drugs.

To investigate whether disrupting endosomal recycling might be a novel strategy for treating glioblastoma, we set out to determine if the endosomal recycling pathway is dysregulated in this cancer type. Using publicly available genomic, transcriptomic and proteomic datasets we demonstrate that the expression of key regulators of endosomal recycling are frequently altered in GBM, and are linked to poorer survival. These findings suggest that targeting the endosomal recycling pathway in GBM is worthy of further investigation.

## 2 Methods

### 2.1 Samples and datasets

The mRNA expression data and corresponding clinical information for 527 glioblastoma (GBM) tumours and 10 non-tumour brain tissues from the TCGA cohort, and 219 GBM and 28 non-tumour samples from the REMBRANDT cohort were acquired from The Cancer Genome Atlas (TCGA). The gene expression data of 54 tissues from 948 donors was downloaded from the Genotype-Tissue Expression (GTEx) project (dbGaP Accession phs000424. v8. p2).

All statistical analyses were carried out using R 4.2.2 software. Endosomal recycling genes were categorized into 3 groups based on their median gene expression level. Group 1: brain median gene expression difference with the non-brain tissues log [fold change (FC)] < 1. Group 2: brain median gene expression in brain was 1 log [fold change (FC)] or greater higher than the median expression in non-brain tissues. Group 3: median gene expression in brain was 1 log [fold change (FC)] or more lower than the median expression in non-brain tissues.

### 2.2 Identification of differentially expressed genes

Gene expression in non-tumour brain and GBM samples were analysed using the edgeR package in R, and differentially expressed genes were identified with the following criteria: [log2 fold-change (FC)] > 0.5 and P < 0.05 [14]. Gene expression and survival data for individual genes were imported from http://gliovis.bioinfo.cnio.es/into GraphPad Prism v.10, which was used to generate the Kaplan-Meier curves.

The mass spectrometry proteomics dataset PXD014606 [15] was downloaded from the PRIDE database repository. Quantification of up- and downregulated endosomal recycling components was performed in Microsoft Excel, by calculating the log2 ratios of the individual endosomal recycling regulators in glioma biopsies compared to control non-tumour brain tissue. Box plots displaying the median, the first and third quartile, and whiskers indicating the minima and maxima were generated in GraphPad Prism v.10.

### 2.3 Prognosis analysis

Overall survival (OS) analysis was performed based on Kaplan–Meier curves to explore the prognostic value of potential GBM-associated endosomal recycling genes. Log-rank P-value <0.05 was considered significant. In addition, univariate Cox regression analysis of gene expression level was performed using the “survival” R package and used to screen for prognosis-related genes in GBM (p < 0.05 was considered significant).

An optimal risk scoring model based on the prognostic potential of the endosomal recycling genes was calculated using a Least Absolute Shrinkage and Selection Operator (LASSO) regression analysis. The “glmnet” R package was used and a risk score was applied to all glioblastoma samples in the dataset and the samples were separated into low- and high-risk groups using the median as a cut-off. A Cox regression model incorporating age and the log-rank test were used to assess overall survival (OS) of the two groups in the whole dataset.

Receiver operating characteristic (ROC) curves to evaluate the effectiveness and accuracy of the risk score in predicting the prognosis were generated by the “timeROC” R package.

## 3 Results

### 3.1 Characterisation of the gene expression profile of endosomal recycling regulators

**Fig. 1:**
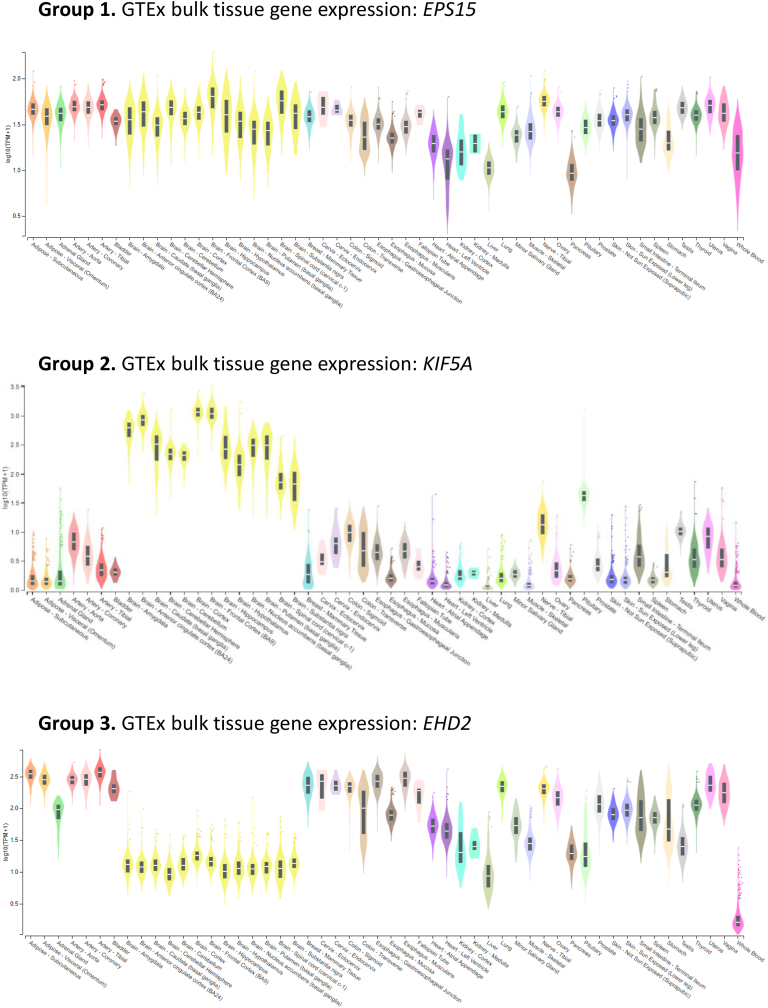
**RNA expression of endosomal recycling genes in normal tissues.** Violin plots of GTEx bulk tissue gene expression for representative endosomal recycling genes from groups 1, 2 and 3. Horizontal lines indicate median gene expression.

We have taken a computational approach to determine if the endosomal recycling pathway is altered in glioblastoma. To this end, we compiled a list of 71 genes that have been reported in the literature to regulate the endosomal recycling pathway (Table 1). The bulk expression for each gene was analysed using the Genotype-Tissue Expression (GTEx) Portal to determine their tissue-specific gene expression profiles [16]. This allowed us to divide the genes into three groups based on their transcript levels; Group 1: ubiquitously expressed (less than 1 log-fold difference between the median expression in brain tissue and the median expression in all other tissues, *n* = 41); Group 2: preferentially expressed in the brain (the median expression in brain tissue is 1 log-fold or more greater than the median expression in all other tissues, *n* = 12); Group 3: not expressed in the brain (1 log-fold or more lower median expression in brain tissue compared to the median expression in all other tissues, *n* = 21). We chose *EPS15*, *KIF5A* and *EHD2* as representatives of groups 1, 2 and 3, respectively (Fig. 1).

**Table 1:** The list of endosomal recycling regulator genes analysed in this study.

Uniprot Gene Name	Full Name	Accession No.	TCGA_GBM LogFC	REMBRANDT LogFC
AP2A1	Adaptor Related Protein Complex 2 Subunit Alpha 1	O95782		−0.332
AP2A2	Adaptor Related Protein Complex 2 Subunit Alpha 2	O94973	−1.182	−0.411
ARF1	ADP-ribosylation factor 1	P84077	−0.279	0.122
ARF6	ADP-ribosylation factor 6	P62330	0.801	0.409
ATP9A	ATPase Phospholipid Transporting 9A	O75110	−0.982	−0.604
CAV1	Caveolin 1	Q03135	3.347	2.653
CCDC22	Coiled-Coil Domain Containing 22	O60826	0.073	0.433
CCDC93	Coiled-Coil Domain Containing 93	Q567U6	0.480	0.349
CMTM6	CKLF Like MARVEL Transmembrane Domain Containing 6	Q9NX76	1.832	0.739
COMMD1	COMM Domain Containing 1	Q8N668		0.183
COMMD2	COMM Domain Containing 2	Q86X83		0.859
COMMD3	COMM Domain Containing 3	Q9UBI1	0.153	0.007
COMMD4	COMM Domain Containing 4	Q9H0A8	0.834	0.752
COMMD5	COMM Domain Containing 5	Q9GZQ3		0.553
COMMD6	COMM Domain Containing 6	Q7Z4G1		0.291
COMMD7	COMM Domain Containing 7	Q86VX2		−0.015
COMMD8	COMM Domain Containing 8	Q9NX08	0.768	0.228
DAB2	DAB Adaptor Protein 2	P98082	1.840	1.420
DNM1	Dynamin-1	Q05193	−2.332	−1.979
EHD1	EH Domain Containing 1	Q9H4M9	0.257	0.485
EHD2	EH Domain Containing 2	Q9NZN4	1.504	0.900
EHD3	EH Domain Containing 3	Q9NZN3	−2.795	−0.462
EHD4	EH Domain Containing 4	Q9H223	1.452	0.235
EPHA2	EPH Receptor A2	P29317	0.752	0.598
EPS15	Epidermal growth factor receptor substrate 15	P42566	−1.070	−0.887
FER	FER Tyrosine Kinase	P16591	0.562	0.215
FLNB	Filamin B	O75369	0.456	0.412
GOLM1	Golgi Membrane Protein 1	Q8NBJ4	0.633	0.375
KDELR1	KDEL Endoplasmic Reticulum Protein Retention Receptor 1	P24390	1.232	0.939
KIF13B	Kinesin Family Member 13B	Q9NQT8	0.305	0.097
KIF5A	Kinesin Family Member 5A	Q12840	−3.331	−1.131
KIF5B	Kinesin Family Member 5B	P33176	0.117	−0.067
KIF5C	Kinesin Family Member 5C	O60282	−2.481	−1.351
LRRK1	Leucine Rich Repeat Kinase 1	Q38SD2	−0.807	−0.146
LRRK2	Leucine Rich Repeat Kinase 2	Q5S007		0.029
MICALL1	MICAL Like 1	D3ZQL6	0.900	0.599
MYO5A	Unconventional myosin-Va	Q9Y4I1	−0.862	−0.951
MYO5B	Unconventional myosin-Vb	Q9ULV0		0.130
NUMB	Protein numb homolog	P49757	0.354	0.274
RAB10	RAB10, Member RAS Oncogene Family	P61026		0.477
RAB11A	RAB11A, Member RAS Oncogene Family	P62491	0.184	−0.004
RAB11B	RAB11B, Member RAS Oncogene Family	Q15907	−0.079	−0.012
RAB11FIP1	RAB11 Family Interacting Protein 1	Q6WKZ4	0.763	−0.274
RAB11FIP2	RAB11 Family Interacting Protein 2	Q7L804	−1.554	−1.068
RAB11FIP3	RAB11 Family Interacting Protein 3	O75154	−0.441	0.309
RAB11FIP4	RAB11 Family Interacting Protein 4	Q86YS3	−0.206	−1.252
RAB11FIP5	RAB11 Family Interacting Protein 5	Q9BXF6	−1.570	−0.504
RAB14	RAB14, Member RAS Oncogene Family	P61106	0.305	−0.067
RAB25	RAB25, Member RAS Oncogene Family	P57735	1.158	−0.084
RAB35	RAB35, Member RAS Oncogene Family	Q15286	−0.252	0.008
RAB4A	Ras-related protein Rab-4A	P20338	0.242	0.042
RAB4B	Ras-related protein Rab-4B	P61018	−0.897	−0.278
RAB8A	Ras-related protein Rab-8A	P61006	1.295	0.870
RABB8	Ras-related protein Rab-8B	Q92930	0.984	0.440
RUFY1	RUN and FYVE domain-containing protein 1	Q96T51	0.637	0.531
RUFY2	RUN and FYVE domain-containing protein 2	Q8WXA3	−0.059	−0.241
SH3GL2	Endophilin A1	Q99962		0.310
SNX1	Sorting Nexin 1	Q13596	0.588	0.308
SNX17	Sorting Nexin 17	Q15036	0.028	0.371
SNX2	Sorting Nexin 27	Q96L92	0.570	−0.127
SNX25	Sorting Nexin 25	Q9H3E2		−0.387
SNX27	Sorting Nexin 27	Q96L92	−0.542	−0.425
SORL1	Sortilin Related Receptor 1	Q92673	−0.401	−0.008
STON2	Stonin 2	Q8WXE9		−0.139
STX12	Syntaxin 12	Q86Y82	−0.066	0.083
TBC1D2	TBC1 Domain Family Member 2	Q9BYX2	0.174	−0.676
TBC1D9	TBC1 Domain Family Member 9	Q6ZT07	−1.309	−1.365
VAMP2	Vesicle Associated Membrane Protein 2	P63027	−2.178	−0.152
VPS26	VPS26 Retromer Complex Component	O75436	−0.350	−0.060
VPS29	VPS29 Retromer Complex Component	Q9UBQ0		0.081
VPS35	VPS35 Retromer Complex Component	Q96QK1	−0.400	−0.007

### 3.2 Endosomal recycling regulator expression is dysregulated in GBM

**Fig. 2:**
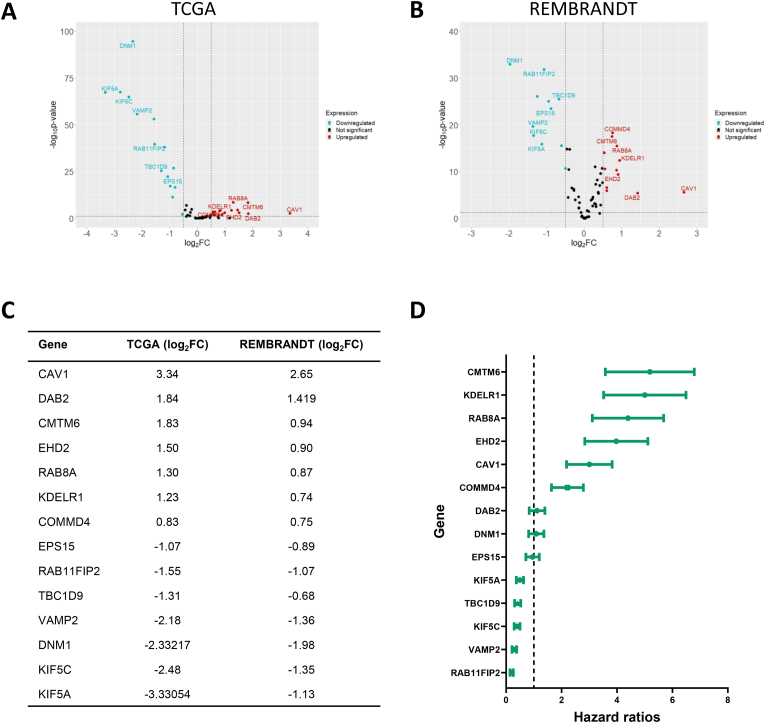
**Differentially expressed endosomal recycling genes.** Volcano plots of differentially expressed endosomal recycling genes in the TCGA (**A**) and REMBRANDT (**B**) GBM cohorts. Red dots are significantly upregulated genes, blue dots are significantly downregulated (*p* value < 0.05 and log_2_FC > 0.5). **C** List of the endosomal recycling genes that are differentially regulated in both the TCGA and REMBRANDT datasets. LogFC values indicated. **D** Forest plots of the differentially expressed endosomal recycling genes indicating their relationship with overall survival. Indicated are hazard ratio and 95% confidence interval.

Next, to determine if endosomal recycling genes are differentially expressed between glioblastoma and non-tumour brain tissue, we analysed their expression in the TCGA_GBM microarray expression dataset, which contains 528 GBM samples and 10 non-tumour brain samples. We also analysed the same genes in an independent brain cancer gene expression dataset, the Repository for Molecular Brain Neoplasia (REMBRANDT) collection, which includes 219 GBM and 28 non-tumour samples and uses the same microarray gene expression chip as the TCGA dataset [17]. The expression of 16 endosomal recycling regulator genes were upregulated and 14 were downregulated in the TCGA dataset (Fig. 2A). Similarly, 12 genes were significantly upregulated and 11 downregulated in the REMBRANDT dataset (Fig. 2B). Comparison of the results from both datasets revealed that 7 of the upregulated genes and 7 of the downregulated genes were shared between both datasets, thus ∼ 19% of the endosomal recycling genes were consistently differentially expressed in GBM (Fig. 2C). These were selected for further analysis. We performed univariate cox regression analysis to reveal the relationship between their expression and prognosis (Fig. 2D). Six genes, *CMTM6, KDELR1, RAB8A, EHD2, CAV1* and *COMMD4,* were identified as risk factors (hazard ratio >1). Five genes, *KIF5A, TBC1D9, KIF5C, VAMP2* and *RAB11FIP2,* had a protective effect (hazard ratio <1).

**Fig. 3:**
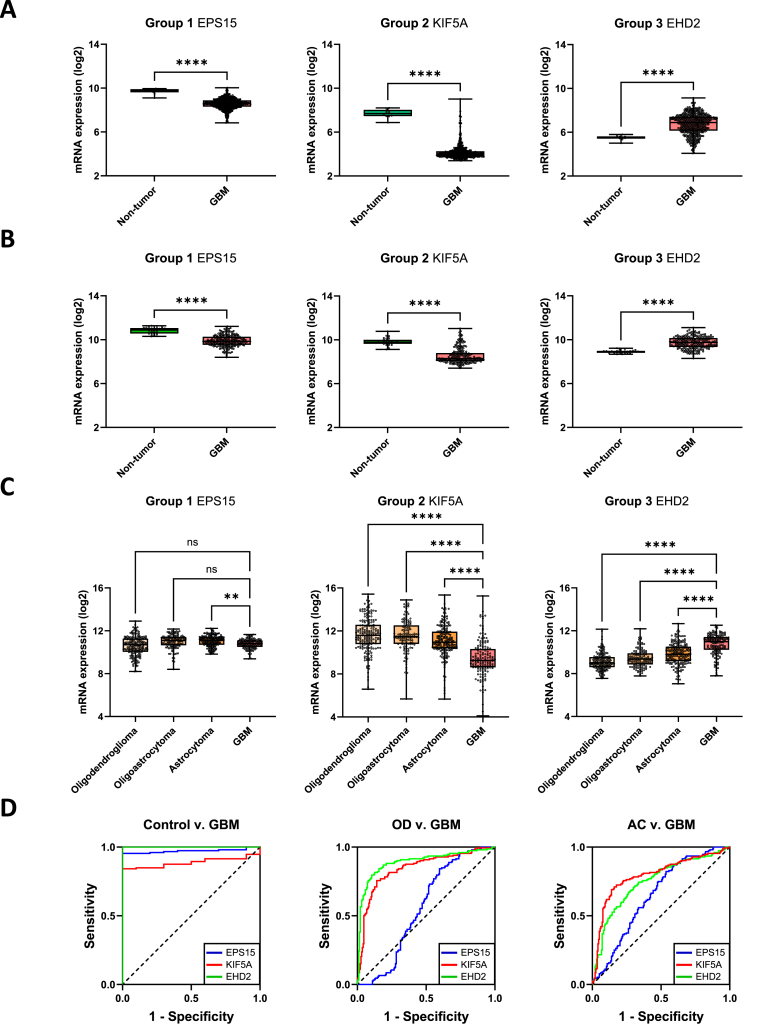
**Endosomal recycling gene expression in glioma.** Comparison of the expression of the representative differentially expressed endosomal recycling genes in the TCGA (**A**) and REMBRANDT (**B**) cohorts. **C** Expression of endosomal recycling genes in the TCGA_GBMLGG dataset. **D** Receiver operating characteristic (ROC) curves indicating the accuracy of gene expression at distinguishing glioma subtype.

Next, we looked at a representative example from each group in more detail (*EPS15* from group 1, *KIF5A* from group 2, and *EHD2* from group 3). The mRNA expression of *EPS15* and *KIF5A* was significantly downregulated in both the TCGA and REMBRANDT glioblastoma datasets, and *EHD2* was upregulated in both datasets (Fig. 3A and B). GBM is a form of glioma, which accounts for 78% of all malignant brain tumours. Gliomas can be divided into four grades. Low-grade glioma (grades 1 and 2) includes oligodendrogliomas and diffuse astrocytomas. Anaplastic astrocytoma and GBM are high-grade gliomas (grades 3 and 4). By definition, GBM is always a grade 4 glioma. There is a statistically significant downregulation in the expression of *EHD1* between astrocytoma and GBM, but no difference between GBM and the two low-grade gliomas (Fig. 3C). In contrast, *KIF5A* and *EHD2* displayed differential expression between GBM and all other gliomas (Fig. 3C). Receiver operating characteristic (ROC) curve analysis of each of these differentially expressed genes revealed that all had high accuracy in differentiating non-tumour brain tissue from GBM, and *KIF5A* and *EHD2* could also accurately distinguish oligodendroglioma and astrocytoma from GBM (Fig. 3D–Table 2).

**Table 2:** Area under the curve (AUC) values for the indicated genes.

	Area under the curve (AUC)		
	Non-tumour v GBM	OD v GBM	AC v GBM
EPS15	0.9717	0.5592	0.653
KIF5A	0.8875	0.8438	0.7959
EHD2	1	0.8907	0.7501

**Fig. 4:**
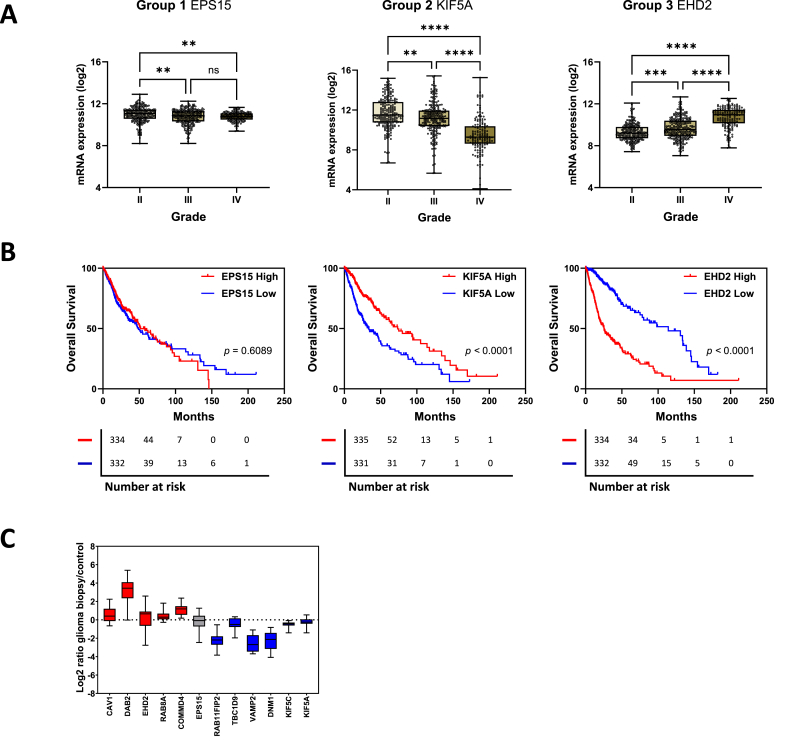
**The prognostic value of endosomal recycling genes. A***KIF5A* and *EHD2* differential expression correlates with tumour grade. **B** Kaplan-Meier analyses were performed based on the expression levels of the representative DEGs in the TCGA_GBMLGG cohort (see Fig. S1 for KM plots from REMBRANDT collection). Median cutoff. **C** Quantitative proteomic analysis of differentially expressed endosomal recycling genes. Proteomic quantiﬁcation of up- (red) and downregulated (blue) endosomal recycling regulators. Plot shows the Log2 ratio of the individual proteins in glioma biopsies compared to control. Box and whisker plot shows the median, the minima and maxima, and the ﬁrst and third quartile.

There was a strong negative correlation between *KIF5A* mRNA expression and tumour grade, and a strong positive correlation between *EHD2* expression and grade (Fig. 4A). *EPS15* did not display the same level of correlation. To determine if there is a link between gene expression and survival, the TCGA_GBMLGG dataset was divided into high- and low-gene expression cohorts (median cutoff) for each gene, and Kaplan-Meier survival curves plotted. There was no difference in survival between the high- and low- *EPS15* expression cohorts. However, the high *KIF5A* expression cohort had considerably better prognosis, while the high *EHD2* expression cohort had much poorer overall survival (Fig. 4B). Almost identical patterns were observed in the REMBRANDT collection (Fig. S1).

Recently, Buser et al. performed an unbiased quantitative proteomics analysis of human glioma [15]. They used mass spectrometry to compare protein expression levels from 4 human glioma biopsies with 4 control human brain biopsies. We analysed the protein expression of 12 of the 14 differentially expressed endosomal recycling regulators in this glioma proteomics inventory (CMTM6 and KDELR1 were missing from this dataset). The protein expression levels of these endosomal recycling regulators closely matched their transcriptomic expression (Fig. 4C).

### 3.3 Construction of a prognostic model with endosomal recycling genes

**Fig. 5:**
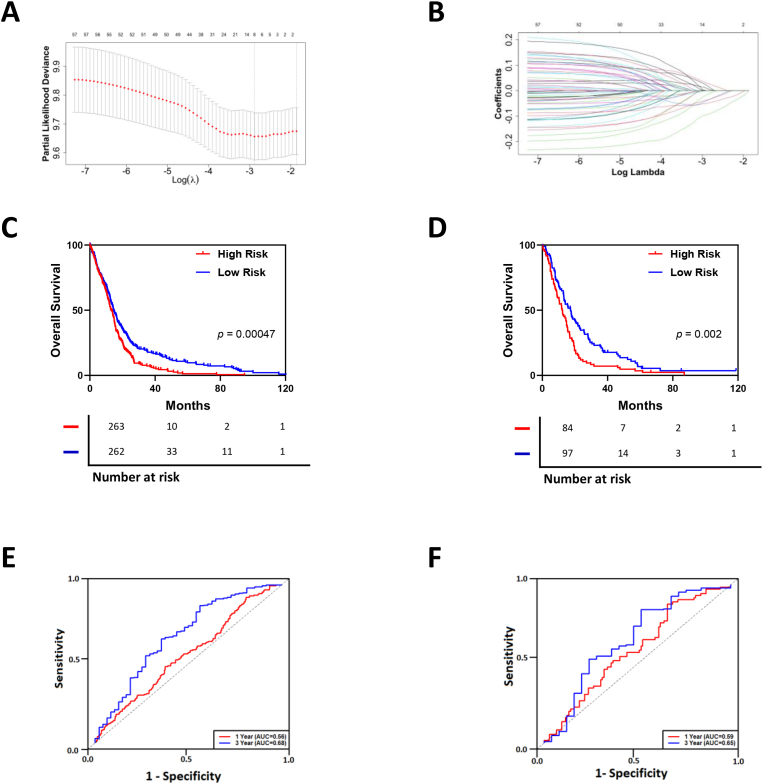
**Construction of a prognostic endosomal recycling gene signature. A.** Determination of the optimal parameter (lambda) in the LASSO model of the endosomal recycling genes in GBM. **B** LASSO coefficient profiles of endosomal recycling genes in glioma. A coefficient profile plot was generated against the log (lambda) sequence. **C** Kaplan–Meier survival analysis of the 8-gene endosomal recycling gene signature from the test TCGA_GBM dataset, comparing OS between the high-risk and low-risk groups. **D** Validation of the endosomal recycling gene signature in the REMBRANDT cohort. **E, F** ROC curve analyses for the test (TCGA_GBM) and validation (REMBRANDT) sets, respectively.

Finally, we used a LASSO Cox regression model to overcome overfitting and to identify the most robust genes for the construction of a gene signature that could be beneficial for GBM prognosis (Fig. 5A and B). We used the data from the TCGA_GBM database as a training set, and the REMBRANDT GBM data as a validation set. We consequently identified an 8-gene signature that was significantly correlated with OS in GBM (Fig. 5C). Of these, *CMTM6, AP2A2,* and *DNM1* showed a positive correlation with the risk score while *KIF5B, ATP9A, COMMD3, RAB11B and VPS26A* had negative correlations (Table 3). Based on these, we determined the corresponding risk scores of GBM patients, and found that patients with high-risk scores had significantly shorter survival times than those with low-risk scores, in both datasets (Fig. 5C and D). The predictive power of the gene-set was confirmed by ROC curve analysis (Fig. 5E and F).

**Table 3:** Multivariate Cox analysis among the endosmal recycling regulators in the training cohort.

The coefficients of the calculation formula for the risk score				
Gene	Coefficient	Hazard ratio	95% CI low	95% CI high
AP2A2	0.008888426	0.6415	0.4964	0.8289
ATP9A	−0.038885969	0.223	0.1671	0.2976
CMTM6	0.029264015	5.020387	3.670756	6.866238
COMMD3	−0.093653346	1.275536	0.985687	1.650616
DNM1	0.019784966	1.063787	0.82449	1.372538
KIF5B	−0.036561744	0.277607	0.209566	0.367738
RAB11B	−0.016118567	0.765313	0.592799	0.988032
VPS26A	−0.04950922	0.399773	0.305305	0.523472

## 4 Discussion

Glioblastoma is among the most difficult cancers to treat, with one of the lowest 5-year survival rates. It has high rates of recurrence and resistance to treatment, largely due to its cellular heterogeneity and extensive tumour invasion into surrounding brain tissue, making complete surgical resection impossible [3]. Furthermore, the recurrent tumour often has a more malignant phenotype [6]. To combat these characteristics of GBM it is important to gain a thorough understanding of the mechanisms that lead to resistance, with a view to identifying common features that may be targeted for therapeutic intervention. To that end, we set out to investigate whether the endosomal recycling pathway, an intracellular membrane trafficking pathway that is dysregulated in other cancers, is also disrupted in glioblastoma [18].

The repertoire of proteins that a cell maintains at the plasma membrane determines how it interacts with neighbouring cells, which nutrients it can internalise, and how it responds to extracellular signals. These cell surface proteins are continuously internalised into early endosomes by a process called endocytosis. From here they are either sorted into late endosomes and lysosomes, where they are degraded, or returned to the plasma membrane. Disruption of this process has been associated with numerous neurological and metabolic diseases, as well as many cancers. Bacteria and viruses also often hijack parts of the membrane trafficking machinery during their life cycle [9]. The endosomal recycling pathway mediates the transport of internalised cell surface proteins back to the plasma membrane, either directly from early endosomes, or indirectly via an endosomal recycling compartment [19]. Dozens of proteins are known to regulate this trafficking pathway, and the expression of many of these is altered in breast, ovarian, head and neck squamous cell carcinoma, and lung cancers [[20], [21], [22], [23], [24]].

Recently, Buser et al. used an unbiased quantitative proteomic analysis to find that multiple components of the endocytosis pathway were massively downregulated in human gliomas compared to non-tumour brain tissue. They showed that this resulted in increased cell surface receptor levels and persistent receptor tyrosine kinase signalling from the cell surface. They proposed that defective endocytosis creates a selective advantage for glioma tumour progression [15]. The involvement of a defective endocytic pathway in the development of glioma has also been reported by Wang et al. who used a bioinformatics approach to generate a signature of endocytosis genes that could be used to stratify low-grade gliomas into high- and low-risk groups [25].

Since dysregulated endosomal recycling can increase the aggressiveness of other cancers by upregulating receptor signalling and promoting migration and invasion, we used cancer genomics to determine if this pathway might also be aberrantly activated in GBM.

40–60% of glioblastomas carry mutations or increased copy numbers of the *EGFR* gene [4]. Recycling is associated with prolonged signalling, while degradation attenuates signalling [26]. Mechanistically it can be envisaged that upregulation of the endosomal recycling pathway would further increase the density of EGFR (and other RTKs) on the cell surface, by shifting the balance between recycling and degradation towards recycling. This would result in an amplification of downstream signal transduction, such as the ERK and Akt pathways, and promote tumour development and invasion into the surrounding tissue. Indeed, a recent study using cultured GBM cells has reported that upregulated endosomal recycling does indeed lead to increased EGFR at the cell surface and sustained proliferative signalling [27].

However, the link between dysregulation of endosomal recycling and cancer progression is complicated by the fact that, depending on the cancer type (or even subtype), the same gene can play a tumour promoting or a tumour suppressive role. For example, Rab25 has been reported to act as an oncogene in breast, ovarian, lung, GBM and gastric cancer and as a tumour suppressor in colon, oesophageal, head and neck and triple-negative breast cancer [28]. Similarly, both overexpression and downregulation of RCP (Rab11FIP1) have been reported to promote breast tumour progression [20,29,30].

Gene expression data of normal brain tissue and GBM biopsies, acquired from publicly available cancer genomics databases, has been used to study the underlying mechanisms of GBM progression, to construct prognostic signatures and to identify novel drug targets [25,[31], [32], [33]]. We used a similar approach to gain a better understanding of the pathophysiological role that endosomal recycling may play in GBM. We first examined the expression pattern of 71 genes that encode regulators of this trafficking pathway. We identified 14 genes that were differentially expressed in two independent glioblastoma transcriptomics datasets. High expression of 6 of these were associated with decreased OS, while high expression of 5 had a protective effect. Further, we showed that the expression levels of a selection of these genes could accurately distinguish low-grade gliomas from high-grade gliomas, and their differential expression correlated with tumour grade. Using the Buser et al. proteomics dataset [15], we confirmed that the differential gene expression we observed from the transcriptomics datasets correlated closely with the protein expression levels.

To further analyze the informativeness of endosomal recycling regulators for prognosis, a risk-scoring model of GBM was used to construct an 8-gene endosomal recycling prognostic signature based on their expression. Kaplan-Meier survival curves and ROC analysis in training and validation datasets confirmed the effectiveness of this model at stratifying GBM into higher and lower risk groups.

It is well established that dysregulated endosomal recycling promotes tumour progression in other cancers. To our knowledge, this is the first time that the endosomal recycling pathway has been analysed systematically in GBM. Our findings show that key components of the endocytic recycling machinery are aberrantly expressed in glioblastoma and suggest that they contribute to the aggressiveness of this cancer. Thus, inhibition of this pathway with small molecule inhibitors may have therapeutic benefit. We have previously shown that chemical and genetic inhibition of endosomal recycling reduces the aggressiveness of lung cancer cells [34]. There are a number of recycling inhibitors available, and we have recently reported that small molecule endosomal recycling inhibitors (ERIs) synergise with HER2-targeting therapies in drug-sensitive and drug-resistant breast cancer cells [12]. Unpublished work from our laboratory using GBM cell lines has found that these ERIs also synergise with the EGFR-targeting TKIs gefitinib and osimertinib.

The main limitation of this study is that the data comes entirely from publicly available databases and the involvement of the dysregulated endosomal recycling regulators in GBM aggressiveness has not been verified experimentally. Further work will involve modulating the expression of the individual endosomal recycling regulators identified in this study in GBM cell lines and *in vivo* models, to determine their specific roles in RTK trafficking, cell proliferation, and invasion.

## Ethical approval

Not applicable.

## Availability of data and materials

All datasets used are publicly available and can be accessed from TCGA or GlioVis [35].

## Funding

This work was funded by a grant from the School of Biochemistry & Cell Biology, University College Cork, Ireland.

## CRediT authorship contribution statement

**Luke J. Joyce:** Writing – original draft, Visualization, Methodology, Investigation, Conceptualization. **Andrew J. Lindsay:** Writing – original draft, Supervision, Investigation, Funding acquisition.

## Declaration of competing interest

The authors declare that they have no known competing financial interests or personal relationships that could have appeared to influence the work reported in this paper.

## Data Availability

Data will be made available on request.

## References

[1] Grech N., Dalli T., Mizzi S., Meilak L., Calleja N., Zrinzo A. (2020). Rising incidence of glioblastoma multiforme in a well-defined population. Cureus.

[2] Stupp R., Mason W.P., van den Bent M.J., Weller M., Fisher B., Taphoorn M.J.B. (2005). Radiotherapy plus concomitant and adjuvant temozolomide for glioblastoma. N. Engl. J. Med..

[3] Zhang P., Xia Q., Liu L., Li S., Dong L. (2020). Current opinion on molecular characterization for GBM classification in guiding clinical diagnosis, prognosis, and therapy. Front. Mol. Biosci..

[4] McLendon R., Friedman A., Bigner D., Van Meir E.G., Brat D.J., Mastrogianakis G.M. (2008). Comprehensive genomic characterization defines human glioblastoma genes and core pathways. Nature.

[5] Vivanco I., Ian Robins H., Rohle D., Campos C., Grommes C., Nghiemphu P.L. (2012). Differential sensitivity of glioma- versus lung cancer-specific EGFR mutations to EGFR kinase inhibitors. Cancer Discov..

[6] Qin A., Musket A., Musich P.R., Schweitzer J.B., Xie Q. (2021). Receptor tyrosine kinases as druggable targets in glioblastoma: do signaling pathways matter?. Neurooncol Adv.

[7] Liu X., Chen X., Shi L., Shan Q., Cao Q., Yue C. (2019). The third-generation EGFR inhibitor AZD9291 overcomes primary resistance by continuously blocking ERK signaling in glioblastoma. J. Exp. Clin. Cancer Res..

[8] Cardona A.F., Jaramillo-Velásquez D., Ruiz-Patiño A., Polo C., Jiménez E., Hakim F. (2021). Efficacy of osimertinib plus bevacizumab in glioblastoma patients with simultaneous EGFR amplification and EGFRvIII mutation. J. Neuro Oncol..

[9] O'Sullivan M.J., Lindsay A.J. (2020). The endosomal recycling pathway-at the crossroads of the cell. Int. J. Mol. Sci..

[10] Ko M., Makena M.R., Schiapparelli P., Suarez-Meade P., Mekile A.X., Lal B. (2022). The endosomal pH regulator NHE9 is a driver of stemness in glioblastoma. PNAS Nexus.

[11] Cheng C., Tu J., Hu Z., Chen Y., Wang Y., Zhang T. (2022). SREBP2/Rab11s/GLUT1/6 network regulates proliferation and migration of glioblastoma. Pathol. Res. Pract..

[12] Mishra A., Hourigan D., Lindsay A.J. (2022). Inhibition of the endosomal recycling pathway downregulates HER2 activation and overcomes resistance to tyrosine kinase inhibitors in HER2-positive breast cancer. Cancer Lett..

[13] Lindsay A.J., McCaffrey M.W. (2017). Rab coupling protein mediated endosomal recycling of N-cadherin influences cell motility. Oncotarget.

[14] Ritchie M.E., Phipson B., Wu D., Hu Y., Law C.W., Shi W. (2015). Limma powers differential expression analyses for RNA-sequencing and microarray studies. Nucleic Acids Res..

[15] Buser D.P., Ritz M.F., Moes S., Tostado C., Frank S., Spiess M. (2019). Quantitative proteomics reveals reduction of endocytic machinery components in gliomas. EBioMedicine.

[16] Ardlie K.G., DeLuca D.S., Segrè A.V., Sullivan T.J., Young T.R., Gelfand E.T. (2015). The Genotype-Tissue Expression (GTEx) pilot analysis: multitissue gene regulation in humans. Science.

[17] Gusev Y., Bhuvaneshwar K., Song L., Zenklusen J.C., Fine H., Madhavan S. (2018). The REMBRANDT study, a large collection of genomic data from brain cancer patients. Sci. Data.

[18] Goldenring J.R. (2013). A central role for vesicle trafficking in epithelial neoplasia: intracellular highways to carcinogenesis. Nat. Rev. Cancer.

[19] Maxfield F.R., McGraw T.E. (2004). Endocytic recycling. Nat. Rev. Mol. Cell Biol..

[20] Zhang J., Liu X., Datta A., Govindarajan K., Wai L.T., Han J. (2009). RCP is a human breast cancer-promoting gene with Ras-activating function. J. Clin. Invest..

[21] Tavares S., Liv N., Pasolli M., Opdam M., Rätze M.A.K., Saornil M. (2022). FER regulates endosomal recycling and is a predictor for adjuvant taxane benefit in breast cancer. Cell Rep..

[22] Yu J., Feng H., Sang Q., Li F., Chen M., Yu B. (2023). VPS35 promotes cell proliferation via EGFR recycling and enhances EGFR inhibitors response in gastric cancer. EBioMedicine.

[23] Goldenring J.R., Nam K.T. (2011). Rab25 as a tumour suppressor in colon carcinogenesis. Br. J. Cancer.

[24] Cheng K.W., Lahad J.P., Kuo W.L., Lapuk A., Yamada K., Auersperg N. (2004). The RAB25 small GTPase determines aggressiveness of ovarian and breast cancers. Nat Med.

[25] Wang D., Liu S., Wang G. (2021). Establishment of an endocytosis-related prognostic signature for patients with low-grade glioma. Front. Genet..

[26] Tomas A., Futter C.E., Eden E.R. (2014). EGF receptor trafficking: consequences for signaling and cancer. Trends Cell Biol..

[27] Sun C., Zhang Y., Wang Z., Chen J., Zhang J., Gu Y. (2024). TMED2 promotes glioma tumorigenesis by being involved in EGFR recycling transport. Int. J. Biol. Macromol..

[28] Cho K.H., Lee H.Y. (2019). Rab25 and RCP in cancer progression. Arch Pharm. Res. (Seoul).

[29] Boulay P.L., Mitchell L., Turpin J., Huot-Marchand J.É., Lavoie C., Sanguin-Gendreau V. (2016). Rab11-FIP1C is a critical negative regulator in ErbB2-mediated mammary tumor progression. Cancer Res..

[30] Caswell P.T., Chan M., Lindsay A.J., McCaffrey M.W., Boettiger D., Norman J.C. (2008). Rab-coupling protein coordinates recycling of α5β1 integrin and EGFR1 to promote cell migration in 3D microenvironments. JCB (J. Cell Biol.).

[31] Wang S., Liu F., Wang Y., Fan W., Zhao H., Liu L. (2019). Integrated analysis of 34 microarray datasets reveals CBX3 as a diagnostic and prognostic biomarker in glioblastoma. J. Transl. Med..

[32] Mu J., Gong J., Shi M., Zhang Y. (2023). Analysis and validation of aging-related genes in prognosis and immune function of glioblastoma. BMC Med. Genom..

[33] Wang D., Jiang Y., Wang T., Wang Z., Zou F. (2022). Identification of a novel autophagy-related prognostic signature and small molecule drugs for glioblastoma by bioinformatics. BMC Med. Genom..

[34] Lindsay A.J., McCaffrey M.W., Lindsay A.J., McCaffrey M.W. (2016). Rab coupling protein mediated endosomal recycling of N-cadherin influences cell motility. Oncotarget.

[35] Bowman R.L., Wang Q., Carro A., Verhaak R.G.W., Squatrito M. (2017). GlioVis data portal for visualization and analysis of brain tumor expression datasets. Neuro Oncol..

