# Supplementary figures and images for: A systematic computational analysis of the endosomal recycling pathway in glioblastoma
Source: Biochem Biophys Rep. 2024 Apr 11;38:101700. doi: 10.1016/j.bbrep.2024.101700 (PMC11024495; doi:10.1016/j.bbrep.2024.101700)

## Slide 1
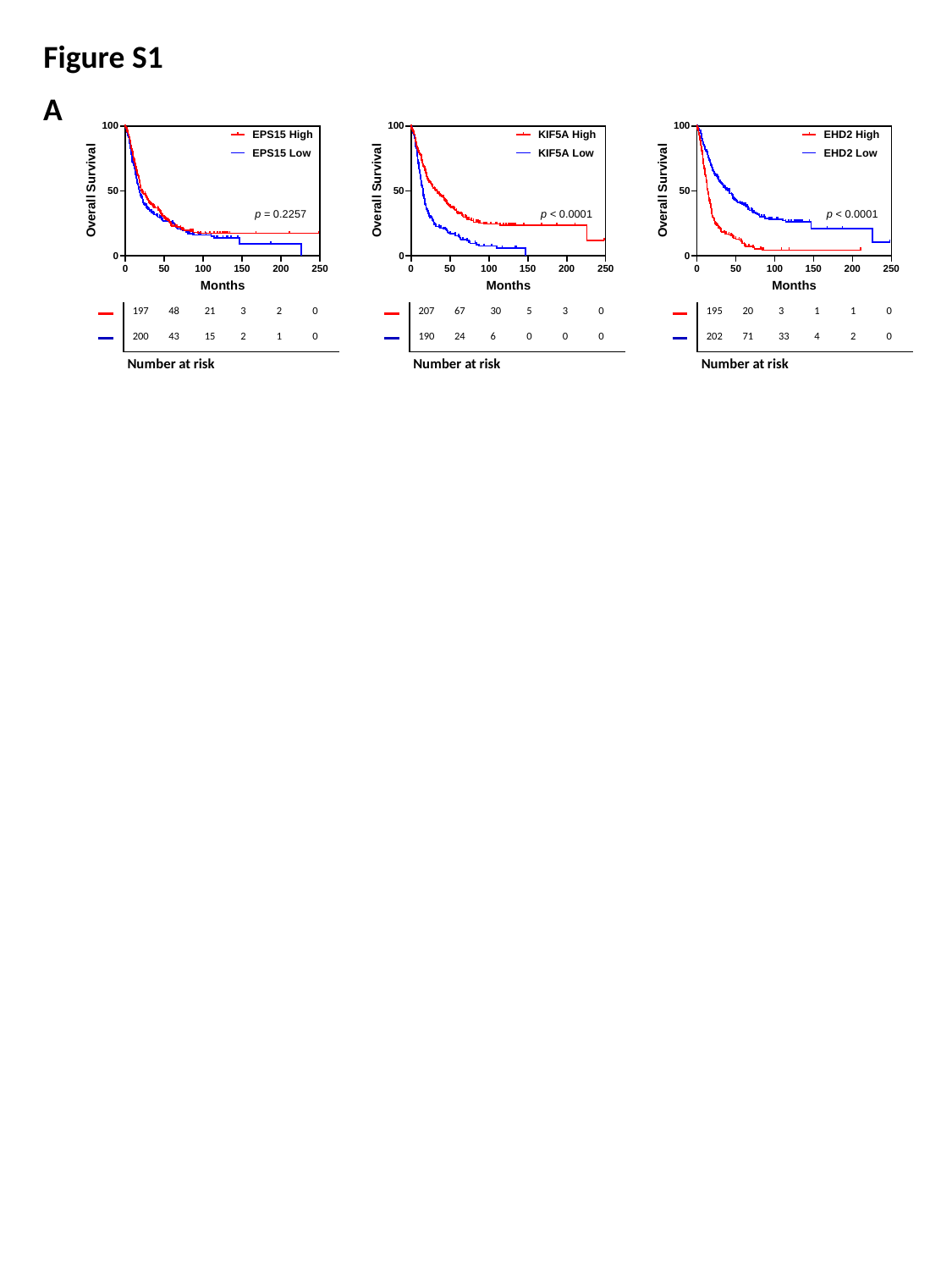

Figure S1
A
| 197 | 48 | 21 | 3 | 2 | 0 |
| --- | --- | --- | --- | --- | --- |
| 200 | 43 | 15 | 2 | 1 | 0 |
| 207 | 67 | 30 | 5 | 3 | 0 |
| --- | --- | --- | --- | --- | --- |
| 190 | 24 | 6 | 0 | 0 | 0 |
| 195 | 20 | 3 | 1 | 1 | 0 |
| --- | --- | --- | --- | --- | --- |
| 202 | 71 | 33 | 4 | 2 | 0 |
Number at risk
Number at risk
Number at risk

Supplement: Multimedia component 1 [file mmc1.pptx]
